# Supplementary material for: Progressive Sarcopenia Correlates with Poor Response and Outcome to Immune Checkpoint Inhibitor Therapy
Source: J Clin Med. 2021 Mar 25;10(7):1361. doi: 10.3390/jcm10071361 (PMC8036296; doi:10.3390/jcm10071361)
Supplement: Supplementary file 1 [file jcm-10-01361-s001.pdf]

Supplementary

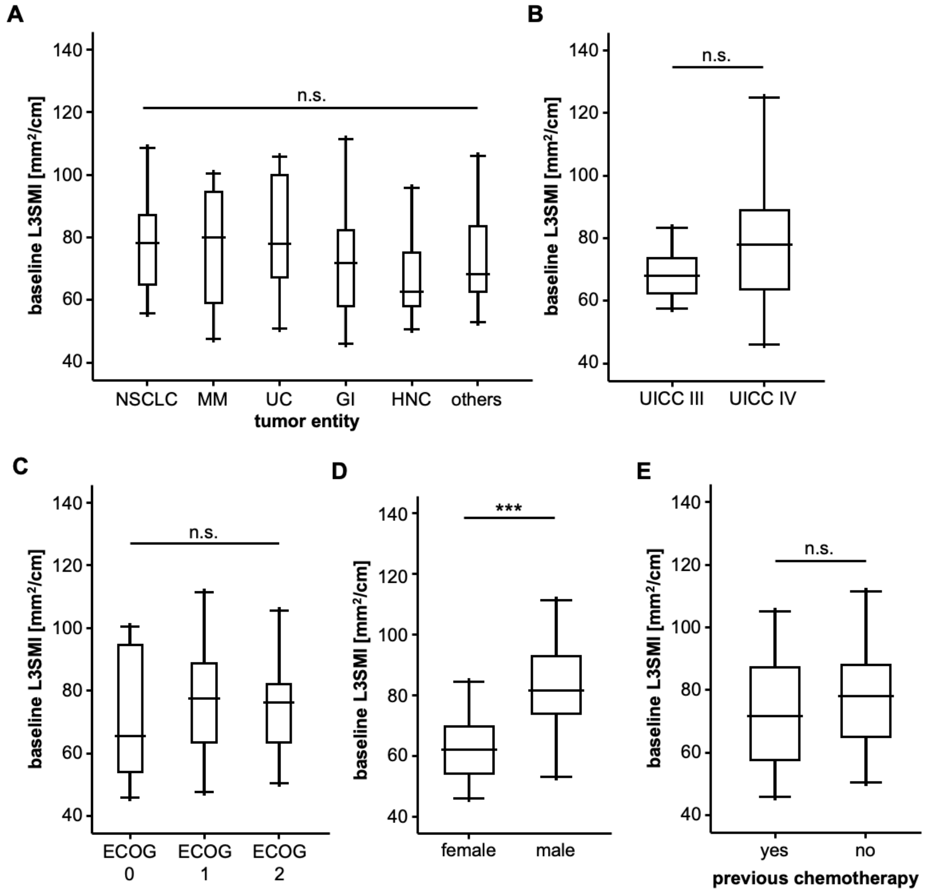

Suppl. Figure 1. The L3SMI as a surrogate for sarcopenia (A) L3SMI values are comparable between patients with different tumor entities (NSCLC: non-small cell lung cancer, MM: malignant melanoma, UC: urothelial carcinoma, GI: gastrointestinal cancer, HNC: head and neck cancer). (B, C) Patients with different UICC tumor stage or ECOG performance status (PS) have comparable L3SMI values. (D) The L3SMI is significantly higher in males compared to female patients. (E) Patient who received previous systemic chemotherapy have comparable L3SMI values than patients who did not.

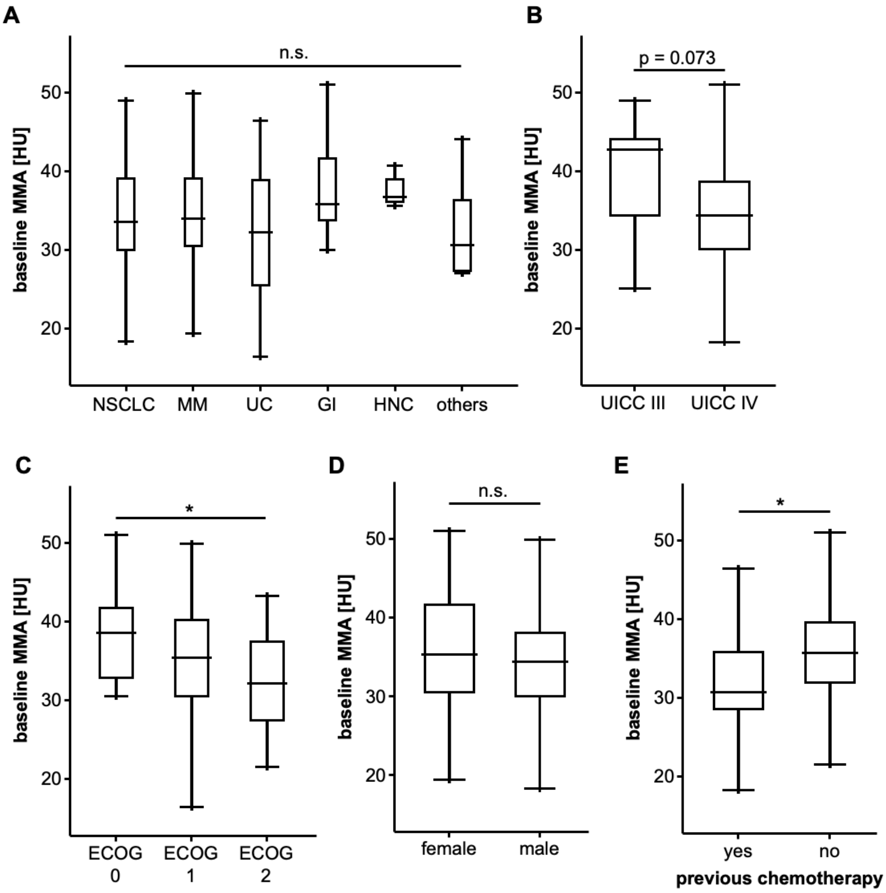

Suppl. Figure 2. The mean skeletal muscule attenuation (MMA) as a surrogate for muscular fat deposition (A) MMA values are comparable between patients with different tumor entities (NSCLC: non-small cell lung cancer, MM: malignant melanoma, UC: urothelial carcinoma, GI: gastrointestinal cancer, HNC: head and neck cancer). (B) Patients with UICC IV tumor stage show a trend towards lower MMA levels compared than UICC III patients. (C) Tumor patients with a poorer ECOG PS have significantly lower MMA values. (D) The MMA is comparable between male and female patients. (E) Patient who received previous systemic chemotherapy have significantly lower MMA values.
